# Data on adult skills formation

**DOI:** 10.1016/j.dib.2019.104953

**Published:** 2019-12-06

**Authors:** Rosario Scandurra, Jorge Calero

**Affiliations:** a Universitat Autònoma de Barcelona, Spain; b Universitat de Barcelona, Spain

**Keywords:** Adult skills, Education, OECD, Structural equation model, PIAAC

## Abstract

This article features supplementary data related to the article “How are adult skills configured?” [1]. The tables show the descriptive statistics of the variables included in the model together with the measurement model and the measure of overall model fit. Moreover, the data article describes the procedures used and can be beneficial for the research community for further research on adult skills. For further information please consult linked data.

**Table:** Specifications Table

Subject area	*Social sciences & Education*
Specific subject area	*Adult skills*
Type of data	*Tables and raw data*
How data were acquired	*The data were retrieved from the OECD webpage*
Data format	*raw*
Parameters for data collection	*Detailed and comparable measures of adult skills*
Description of data collection	*Data collect report on adult skills for respondents between 16 and 65.*
Data source location	*The data is available through OECD webpage and can be downloaded here:*http://www.oecd.org/skills/piaac/publicdataandanalysis/
Data accessibility	*Scandurra, Rosario (2018), “PIAAC_SII”, Mendeley Data, v1*https://doi.org/10.17632/vbtc8f92wc.1
Related research article	*R. Scandurra, J. Calero, How are adult skills configured?, International Journal of Educational Research, (2019).*https://doi.org/10.1016/j.ijer.2019.06.004 [1]

**Table:** 

**Value of the Data** • This article provides additional data and describes the procedure adopted for examining adult skills using PIAAC data • These data can be used as an example for comparative analysis of adult skills which employs Structural Equation Models (SEM). • Readers can benefit of additional data on adult skills configuration in five OECD countries. • These data can be used for further development and research on adult skills.

## 1 Data

This article provides additional data on the configuration of adult skills in five OECD countries. The data contain 142 items for a total of 13,825 respondents aged between 26 and 55 years. These data were used in a recent article [1] based on the theoretical model proposed by Desjardins [2] and further developed in a recent paper [3]. The data were extracted from the Program for the International Assessment of Adult Competencies (PIAAC), released in October 2013 and updated in March 2015. The data are made available on the OECD webpage and were retrieved in April 2017. The first wave^1^ of PIAAC provides direct measures of skills together with rich information on the individual social environment for adults aged between 16 and 65 in 24 countries, mostly OECD members. We employed a Structural Equation Model (SEM) to explore skills configuration for the United States, Japan, Germany, Spain and Denmark. Table 1 provides the information of all the variables included in the model. Table 2 shows the descriptive statistics. Table 3 provides information of the measures of model-fit. Finally, Table 4 details the measurement model.

**Table 1:** Latent and observed variables used in the model.

Latent variables			Observed variables		
Symbol	Label	Abbreviation	Symbol	Description	Type
ξ1		Gender	x1	Gender	dichotomous
ξ2		Age	x2	Age	ordinal
ξ3		Foreign born	x3	Born in country	dichotomous
η1	Family background	F1	y1	Father Higher Education	ordinal
η2	Education	F2	y4	Highest Level of Education	continuous
			y5	Age of obtaining hi. education qual.	ordinal
η3	Use of skills in the workplace	F3	y6	Use of Reading Skills at Work	ordinal
			y7	Use of Numeracy Skills at Work	ordinal
			y8	Use of Writing Skills at Work	ordinal
			y9	Use of Influencing Skills at Work	ordinal
η4	Use of skills at home	F4	y10	Use of Reading Skills at Home	ordinal
			y11	Use of Numeracy Skills at Home	ordinal
			y12	Use of Writing Skills at Home	ordinal
			y13	Use of ICT Skills at Home	ordinal
η5	Literacy proficiency	F5	y14	Plausible value Literacy pvlit1	continuous
			y15	Plausible value Literacy pvlit2	continuous
			y16	Plausible value Literacy pvlit3	continuous
			y17	Plausible value Literacy pvlit4	continuous
			y18	Plausible value Literacy pvlit5	continuous
			y19	Plausible value Literacy pvlit6	continuous
			y20	Plausible value Literacy pvlit7	continuous
			y21	Plausible value Literacy pvlit8	continuous
			y22	Plausible value Literacy pvlit9	continuous
			y23	Plausible value Literacy pvlit10	continuous

**Table 2:** Descriptive statistics.

	Denmark	Germany	Japan	Spain	United States
**Age Recoded 5-Year Groups**					
25-29	10.38	13.58	12.88	13.10	17.42
30-34	14.59	14.28	15.38	16.99	16.43
35-39	16.18	12.95	20.17	18.78	15.81
40-4	20.07	19.43	18.04	19.11	15.85
45-49	20.76	21.07	17.62	18.00	16.68
50-5	18.02	18.70	15.91	14.03	17.80
Missing	0.00	0.00	0.00	0.00	0.00
**Background - Born In Country**					
Yes	75.72	88.27	99.66	86.42	84.43
No	24.19	11.70	0.34	13.58	15.52
Missing	0.09	0.03	0.00	0.00	0.04
**Father Higher Education In 3 Categories**					
ISCED 1, 2, and 3C Short	35.79	9.99	27.27	72.58	21.10
ISCED 3 (Excluding 3C Short) and 4	36.63	52.54	42.84	14.25	44.55
ISCED 5 and 6	26.56	32.45	26.02	11.39	31.54
Missing	1.03	5.01	3.87	1.78	2.81
**Gender**					
Men	50.56	50.87	52.91	53.32	49.26
Women	49.44	49.13	47.09	46.68	50.74
Missing	0.00	0.00	0.00	0.00	0.00
**Age of Obtaining Education (AOE)- Hi. Qualification**					
Aged 15 or Younger	2.37	2.30	3.30	20.56	3.43
Aged 16-19	13.97	29.42	37.22	30.46	27.58
Aged 20-24	32.67	35.06	53.82	29.17	34.97
Aged 25-29	28.96	20.93	3.76	11.80	16.85
Aged 30-34	10.35	7.94	1.14	3.15	8.42
Aged 35 or Older	11.16	3.83	0.65	3.23	7.93
Missing	0.53	0.52	0.11	1.63	0.83
**Index Of Use Of Reading Skills At Work**					
All Zero Response	2.49	3.90	3.76	13.95	3.39
Lowest to 20%	10.10	12.64	12.53	19.89	10.94
More than 20%–40%	14.15	14.73	19.37	17.59	17.51
More than 40%–60%	22.63	19.25	19.83	15.40	19.12
More than 60%–80%	25.31	24.03	19.83	13.65	21.47
More than 80%	25.16	25.42	24.42	19.15	27.46
Missing	0.16	0.03	0.27	0.37	0.12
**Index Of Use Of Numeracy Skills At Work**					
All Zero Response	15.68	15.11	9.57	26.90	12.96
Lowest to 20%	16.52	16.64	15.00	12.88	10.90
More than 20%–40%	15.71	15.32	25.29	14.99	12.14
More than 40%–60%	17.49	15.29	18.91	12.84	17.34
More than 60%–80%	17.58	16.64	15.65	15.47	21.76
More than 80%	16.86	20.96	15.31	16.55	24.86
Missing	0.16	0.03	0.27	0.37	0.04
**Index Of Use Of Writing Skills At Work**					
All Zero Response	7.11	9.16	6.84	23.12	11.60
Lowest to 20%	12.38	12.05	10.03	13.58	12.68
More than 20%–40%	21.66	18.04	14.28	15.44	13.34
More than 40%–60%	22.85	21.48	18.31	13.58	15.57
More than 60%–80%	19.92	20.89	24.08	16.18	21.02
More than 80%	15.93	18.35	26.21	17.74	25.76
Missing	0.16	0.03	0.27	0.37	0.04
**Index Of Use Of Influencing Skills At Work**					
All Zero Response	4.99	9.26	7.14	16.47	4.75
Lowest to 20%	11.10	16.43	20.17	23.45	12.14
More than 20%–40%	15.74	19.67	22.71	16.03	15.98
More than 40%–60%	19.76	22.11	19.03	15.40	16.02
More than 60%–80%	24.41	19.78	17.28	13.58	20.89
More than 80%	23.85	12.67	13.41	14.73	30.10
Missing	0.16	0.07	0.27	0.33	0.12
**Index Of Use Of Reading Skills At Home**					
All Zero Response	0.31	0.14	0.46	1.45	1.07
Lowest to 20%	7.98	8.81	16.45	23.86	8.88
More than 20%–40%	19.64	15.11	27.23	21.82	13.83
More than 40%–60%	27.65	21.41	24.57	18.22	19.61
More than 60%–80%	24.84	26.50	18.88	15.66	22.67
More than 80%	19.45	28.03	12.42	18.96	33.94
Missing	0.12	0.00	0.00	0.04	0.00
**Index Of Use Of Numeracy Skills At Home**					
All Zero Response	5.14	5.57	15.99	14.69	4.42
Lowest to 20%	16.74	16.09	29.70	22.52	10.03
More than 20%–40%	19.45	17.69	24.69	18.70	13.91
More than 40%–60%	22.04	20.89	15.38	14.69	20.23
More than 60%–80%	21.51	23.96	9.68	16.03	25.93
More than 80%	15.02	15.81	4.56	13.36	25.47
Missing	0.09	0.00	0.00	0.00	0.00
**Index Of Use Of Writing Skills At Home**					
All Zero Response	3.40	2.44	7.25	15.81	9.29
Lowest to 20%	21.23	16.99	22.71	28.57	18.37
More than 20%–40%	14.15	10.13	21.08	15.66	10.86
More than 40%–60%	26.47	31.86	24.31	20.15	20.15
More than 60%–80%	18.98	22.08	14.09	9.24	18.04
More than 80%	15.65	16.50	10.56	10.58	23.29
Missing	0.12	0.00	0.00	0.00	0.00
**Index Of Use Of ICT Skills At Home**					
All Zero Response	0.25	0.63	1.79	0.71	0.58
Lowest to 20%	10.44	15.46	32.66	16.03	10.57
More than 20%–40%	14.59	17.37	24.84	16.33	15.52
More than 40%–60%	21.73	19.95	14.01	14.55	17.51
More than 60%–80%	24.41	20.72	6.80	13.36	18.79
More than 80%	24.75	16.33	3.91	13.21	20.85
Missing	3.83	9.54	15.99	25.83	16.18
**Highest Level of Education (years)**					
Mean	13.65	14.44	13.75	12.34	14.24
Standard deviation	2.63	2.72	2.26	3.48	2.92
n	3206	2871	2632	2694	2133
Maximum	22	22	22	22	22
Minimum	3	3	3	3	3
**Index Of Use Of Writing Skills At Home**					
All Zero Response	3.40	2.44	7.25	15.81	9.29
Lowest to 20%	21.23	16.99	22.71	28.57	18.37
More than 20%–40%	14.15	10.13	21.08	15.66	10.86
More than 40%–60%	26.47	31.86	24.31	20.15	20.15
More than 60%–80%	18.98	22.08	14.09	9.24	18.04
More than 80%	15.65	16.50	10.56	10.58	23.29
Missing	0.12	0.00	0.00	0.00	0.00
**Index Of Use Of ICT Skills At Home**					
All Zero Response	0.25	0.63	1.79	0.71	0.58
Lowest to 20%	10.44	15.46	32.66	16.03	10.57
More than 20%–40%	14.59	17.37	24.84	16.33	15.52
More than 40%–60%	21.73	19.95	14.01	14.55	17.51
More than 60%–80%	24.41	20.72	6.80	13.36	18.79
More than 80%	24.75	16.33	3.91	13.21	20.85
Missing	3.83	9.54	15.99	25.83	16.18
**Highest Level of Education (years)**					
Mean	13.65	14.44	13.75	12.34	14.24
Standard deviation	2.63	2.72	2.26	3.48	2.92
n	3206	2871	2632	2694	2133
Maximum	22	22	22	22	22
Minimum	3	3	3	3	3

**Table 3:** Goodness of fit measures for literacy SEM.

	χ2	χ2/df	df	n	RMSEA	90% C.I. RMSEA	CFI	TLI	WRMR
United States	874.081	3.2	273	2421	0.034	0.031–0.036	0.965	0.959	1.102
Japan	828.144	3.03	273	2633	0.031	0.029–0.033	0.973	0.969	1.202
Spain	508.566	1.86	273	2695	0.021	0.018–0.023	0.989	0.987	0.79
Germany	1000.218	3.66	273	2871	0.034	0.032–0.036	0.969	0.964	1.204
Denmark	1125.514	4.12	273	3205	0.035	0.033–0.037	0.968	0.962	1.252

Comparative Fit index (CFI), Tucker – Lewis Index (TLI), Root Mean Square Error of Approximation (RMSEA).

**Table 4:** Measurement model.

		United States			Japan			Spain			Germany			Denmark		
		Estimate	S.E.	P-Value	Estimate	S.E.	P-Value	Estimate	S.E.	P-Value	Estimate	S.E.	P-Value	Estimate	S.E.	P-Value
F1	Fated	0.725	0.004	0.000	0.735	0.004	0.000	0.723	0.004	0.000	0.721	0.003	0.000	0.724	0.003	0.000
F2	Yrsqual	0.995	0.015	0.000	0.909	0.011	0.000	0.933	0.011	0.000	0.973	0.012	0.000	0.951	0.016	0.000
	AOE	0.682	0.015	0.000	0.906	0.013	0.000	0.760	0.013	0.000	0.695	0.014	0.000	0.636	0.016	0.000
F3	Readh_C	0.800	0.014	0.000	0.818	0.016	0.000	0.830	0.013	0.000	0.787	0.015	0.000	0.776	0.014	0.000
	Numh_C	0.718	0.017	0.000	0.707	0.018	0.000	0.655	0.017	0.000	0.718	0.018	0.000	0.702	0.015	0.000
	Writh_C	0.836	0.014	0.000	0.623	0.018	0.000	0.796	0.014	0.000	0.652	0.017	0.000	0.761	0.013	0.000
	Icth_C	0.733	0.017	0.000	0.683	0.020	0.000	0.716	0.018	0.000	0.687	0.019	0.000	0.752	0.015	0.000
F4	Readw_C	0.872	0.014	0.000	0.866	0.013	0.000	0.911	0.010	0.000	0.870	0.011	0.000	0.858	0.013	0.000
	Numw_C	0.688	0.019	0.000	0.716	0.016	0.000	0.708	0.016	0.000	0.753	0.015	0.000	0.712	0.016	0.000
	Writw_C	0.804	0.014	0.000	0.725	0.015	0.000	0.797	0.013	0.000	0.683	0.016	0.000	0.640	0.016	0.000
	Inflw_C	0.608	0.020	0.000	0.642	0.017	0.000	0.687	0.016	0.000	0.721	0.015	0.000	0.641	0.017	0.000
F5	Pvlit1	0.952	0.003	0.000	0.888	0.005	0.000	0.949	0.003	0.000	0.939	0.003	0.000	0.938	0.003	0.000
	Pvlit2	0.947	0.004	0.000	0.894	0.004	0.000	0.939	0.004	0.000	0.938	0.004	0.000	0.938	0.003	0.000
	Pvlit3	0.947	0.004	0.000	0.894	0.004	0.000	0.943	0.003	0.000	0.938	0.003	0.000	0.940	0.003	0.000
	Pvlit4	0.959	0.003	0.000	0.905	0.004	0.000	0.934	0.004	0.000	0.938	0.003	0.000	0.933	0.003	0.000
	Pvlit5	0.951	0.004	0.000	0.893	0.004	0.000	0.946	0.003	0.000	0.932	0.004	0.000	0.939	0.003	0.000
	Pvlit6	0.941	0.004	0.000	0.900	0.004	0.000	0.943	0.003	0.000	0.938	0.003	0.000	0.934	0.003	0.000
	Pvlit7	0.948	0.004	0.000	0.899	0.004	0.000	0.935	0.004	0.000	0.940	0.003	0.000	0.936	0.003	0.000
	Pvlit8	0.948	0.004	0.000	0.900	0.004	0.000	0.942	0.003	0.000	0.942	0.003	0.000	0.940	0.003	0.000
	Pvlit9	0.956	0.003	0.000	0.904	0.004	0.000	0.937	0.003	0.000	0.943	0.003	0.000	0.934	0.003	0.000
	Pvlit10	0.942	0.004	0.000	0.897	0.005	0.000	0.942	0.003	0.000	0.945	0.003	0.000	0.936	0.003	0.000

## 2 Experimental design, materials, and methods

To test the hypothesized relationships between the constructs and to evaluate the theoretical model, we used a Structural Equation Model (SEM). This is a broadly flexible set of statistical techniques, which allows the representation of the constructs of interest and the measurement of the extent to which the data are consistent with a proposed theoretical model.

Table 1 provides a list of the observed and latent variables included in the model. We have measured the four components of skills acquisition as follows: family background using the father's highest level of educational attainment; education using two items (the highest level of education attainment in years and the age of obtaining the highest education qualification); and the practice of skills in the workplace and in the home using four items. We also controlled for age, for being born outside the test country and for gender. For a matter of clarity, Fig. 1 in Ref. [1] shows the path diagram of the model. Finally, the latent construct of literacy and numeracy comprises ten plausible values. The PIAAC framework evaluate literacy and numeracy using 58 and 56 items, respectively, distributed across three main task characteristics (medium, context and aspect) and differentiated between paper and computer-based questions [4]. As in other standardized international educational assessments, PIAAC uses Item-Response Techniques (IRT) to generate ten plausible values of each domain examined.

Table 3 reports the goodness-of-fit measures of the model for numeracy. The estimator selected was the robust weighted least squares (WLSMV), created to deal especially with a combination of ordinal, discrete and continuous data and a small to medium sample size. The estimates were produced using Mplus 7.4. We then scrutinized the modification indices and performed J-Rule using Jrule [5] which implements the method described in Ref. [6]. We performed sensitivity tests including missing data and recoding the zero category of observed indicators in the latent constructs of use of skills into missing data. Bootstrap estimation was performed using 2000 iterations, yielding the same results as the WLSMV estimation.

The model fit indexes were consistent across all countries, with respect to the standard CFI and TLI thresholds (above 0.95). The RMSEA was also below 0.05, pointing to the plausibility of the model. In conclusion, we can reject the null hypothesis of a divergent structure of configuration of skills across the five countries considered.

Therefore, following the standard procedure in the SEM literature, our two-step modelling process included i) a measurement model, describing the way observed variables load onto latent constructs, and ii) a structural model, which estimates the pathways among all the variables, including the latent constructs [7].

Table 4 reports the factor loading of each unobserved latent variable. We performed a confirmatory factor analysis (CFA) of the measurement model specifying the established relationships of the observed variables to the latent constructs. A confirmatory factor analysis (CFA) was performed to check for the consistency of each latent variable (measurement model).

## Footnotes

1 More countries were added in the successive round of PIAAC including over 40 economies.
